# Specialists and Specialities

**Published:** 1861-01

**Authors:** 


### Art. V.—SPECIALISTS AND SPECIALITIES.

A little while ago we were startled from the placid repose of an after-dinner nap by the loud laughter of a friend, one, alas! of too "sensible and nimble lungs." As we opened our eyes widely in blank amazement at the ecstasy of the merriment in which he was indulging, "Listen!" he exclaimed, and for the sake of greater emphasis, he filliped with his fingers the pages of a book he held in his hand. "And pray," we asked, "what work have you laid hold of that so powerfully excites your risibility?" "Oh!" he replied, "this is the new volume of the *Transactions of the Epidemiological Society*. But," he continued, "do not look so ridiculously aghast. It is not the work that is funny in itself; but I have discovered in it a delicious morsel of information. I have stumbled, in fact, over an account of the undoubted prototypes of specialists in physic. Here it is:—

"'The men who practise medicine,' states a Mr. R. Clarke, writing of native doctors, in a most interesting paper on the Topography and Diseases of the Gold Coast of Africa, 'are ranked with the fetish priests. They do not profess to cure disease in general, but devote their attention to the relief of special ailments. Thus they will, when applied to, say whether they have or have not any skill in the treatment of the particular complaint, or as they express it, 'they have no good medicine for its cure.' The consequence is, that there is a vast number of country doctors, each boasting of his skill in the management of his own speciality. Many of them, however, are mere empirics, and deceive their patients by their tricks. Sick persons travel or are carried great distances into the interior to put themselves under a native doctor having a reputation for curing the particular disease they may at the time suffer. The doctor's fee varies according to the rank of the patient; but it is in general a dollar (4*s.* 6*d.* sterling), a fowl, a goat, or a piece of cotton goods, or cutlery,' &c.

"There now, my dear fellow," exclaimed our friend, getting out his words as well as he could amidst a series of half-strangled laughs, "there now, you will perceive that the native physician of the Gold Coast is the very pink of a specialist, and the native physic the perfection of a specialist practice of physic."

"Well," we remarked, with an attempt to maintain an imposing quietude in our voice. "Well, and what follows?"

"Simply this," he responded, "that a specialist doctor is a reversion to an earlier and more degraded type of the doctor; specialist practice of physic to an earlier and more degraded type of physic."

A self-gratified chuckle formed the period to this remark; and our friend evidently plumed himself upon having said a good thing.

But "shall quips and sentences, and these paper bullets of the brain awe a man from the career of his humour?" We trow not. Our friend belongs to a class of persons, far too common both in the profession and out, who, if they see an object defaced by any scum or excrescence, are very apt to conclude that the whole thing is bad and impure. Rarely, or never, does it occur to them to ask whether the offensive spume or out-growth may not be the result of some process of depuration, or an accidental matter disfiguring but not injuring the object at the core. They hasten with averted gaze to the other side of the way, or if, prompted by a zealous horror, they seek to eradicate the evil, they blindly strike both at the true and false—the healthy and the vitiated structure.

If, however, we profess to see deeper into the substance of a particular millstone than some other people, it is but just that, in the first place, we should state what aspect the millstone has to those whose less penetrating glance we, in the wealth of our conceit, commiserate.

And here the authors of the *Memoirs of Bilboquet* come to our help with their impudent but sparkling little sketch, "*Paris Médecin.*" A chapter of the hero's adventures, freely rendered, is very much to our purpose.

Smith has completed the prescribed course of medical study, and stuffed with pathology, saturated with clinical medicine, and perspiring therapeutics at every pore, he presents himself for his final examination. He passes with neither less nor greater credit than hundreds have done before him; and seeking out a promising locality for practice, he settles himself there, and having affixed his name and profession in conspicuous characters to the door of his domicile, he resigns himself to visions of happiness and competence, which he fondly hopes to be in store for him. But days and weeks roll on, and the slowly but surely advancing time frets cruelly away piecemeal the hope which had hitherto buoyed the poor fellow up. He feels himself sinking helplessly and hopelessly beneath a sea of difficulties, when one day, as, in search of distraction, he is wandering in the streets, he is accosted by a quondam fellow-student, Brown. Brown at a glance sees how the land lies with his former companion, and compelling him to enter a stylish little brougham, which appertains to the said Brown, drives him off and deposits him where a good dinner and a good gossip may be had at ease.

Suppose the dinner at an end, and that the two friends are irrigating their feelings with generous wine, when Brown, overflowing with worldly wisdom and kindness to his old crony, thus speaks:—

"My dear Smith, I see that you have attempted physic from the wrong side. Nay, more, you are plainly a century behind the time. If I were to visit your residence, I have no doubt I should find in the reception-room an engraving representing Hippocrates refusing the gifts of Artaxerxes, a skull, and an anatomical drawing by Maclise. I am certain that your good nature has led you to prescribe for every form of sickness upon which you have been consulted; it matters not what the malady might be, whether typhoid fever, phthisis, inflammation of the bowels, neuralgia, gastritis, gastro-enteritis, and so forth. Upon this rock you have been wrecked; upon this rock all young physicians are lost. Now, my dear fellow, let me advise you. Give your engraving to the kitchen-maid, sell the Maclise, and throw the skull into the lumber-room. Any way put it out of sight; for, rest assured, it horrifies patients, particularly when women. Convert your reception-room into a boudoir or drawing-room, and hide the practitioner under the man-of-the-world. The best physician, now-a-days, is he who looks least like a physician. Finally, and mark this well, *become a specialist.*

"The physician of our fathers' days, the encyclopædic physician who grappled with all diseases, is put aside. He is thrust completely into the background, and the public now have faith in specialities and specialists alone. Diseases of the ears, of the eyes, of the nose, of the mouth, and a host of other affections constitute specialities. Each member, each organ of the body has, indeed, its diseases, and consequently its physician, who undertakes to cure them radically; and the physician thus practising, is designated in our new medical phraseology a *specialist.*

"All the specialities are already seized hold of, but that does not prevent new ones being discovered every day. I have discovered a superb one which I have kept back in case of need, but, for the sake of old times, I will give it up to you; it is the Speciality of the Great Toe.

"Treatment of diseases of the Great Toe! In-growth of the nail, bunions, corns, hard and soft—Jove! what a prospect! This treatment includes maladies so lucrative, that it is needful to pluck it from the hands of the ignorant quacks who now practise it. It is time that true medicine executed justice upon empirics and charlatans, and took possession itself of the patients of pedicures. Do not hesitate; put faith in me; throw yourself at once into this path, and adopt frankly the speciality of the Great Toe."

"But," interposed Smith, "what is your speciality?"

"PINGUIFACTURE," answered Brown.

"And what is that?"

"The art," responded the worthy advocate of specialities, "of fattening people at will; of diffusing the adipose matter, vulgarly called fat, over the body in general, or upon any given point of it —upon the arms, the legs, the breast, the shoulders, in short, wherever it may be wished. Thanks to my speciality, crinoline need no longer be worn, and suffering humanity may be rid of it. But not only have I discovered a means of fattening people, but also of causing them to become lean. My speciality then, as you will see, is a double one, and addresses itself to two very numerous classes—the fat and the lean. I do not lack patients, particularly ladies, because stoutness and leanness are the two plagues of the fair sex. The hour for consultation will quickly be here. You shall go with me and witness for yourself my waiting-room crowded, and the court-yard of my house full of carriages."

Brown had not exaggerated. Smith was fain to admit the success of Pinguifacture. The thick shades of old-fashioned physic, which had so long blinded his eyes, were dissipated, and he became a specialist.

Now straining off the exaggeration of this sketch, we shall find that it represents pretty accurately the feeling of large sections of the public and the profession concerning specialists and specialities. A speciality is looked upon by the individuals composing these sections as the last resort of a needy man, or the resource of a shrewd, bold man to push his way rapidly to competence or public fame. That there have been and are specialists who have become such under the influence of these motives we admit; but that the motives constitute the essence of specialist practice we utterly deny. Specialists of this class are but parasitical growths upon a sound stock, disfiguring and masking its noble proportions in the eyes of the negligent gazer, but, although they may fret it, not a whit detracting from its vitality. True specialists are the chief workers in the advancement of medical science; true specialities are the life's blood of medical theory and practice.

Every medical man of active thought is a specialist. Before he has ended his student-life, before he has entered upon the practice of his profession, he has, either from accident or by choice, fixed his mind more particularly on some one or other of the many questions which are included in the theory and practice of physic. To this question he pins his faith of ultimate fame or success. It may be a puny question of little comparative value, yet it suffices to stir up to greater activity, and keep in action the mental powers of its pursuer. Think of the dead monotony, of the repulsive stagnancy of that man's mind who emerges from the trammels of student-life, with an average acquaintance with the many subjects which it has been needful for him to study, but with no greater care for any one, or for any part of any one of those subjects than another. Think you that he will, or that he would if he could (unless perchance he possesses the talent of an Alexander von Humboldt), keep himself abreast of the progress of the different branches of science entering into his preliminary studies? The notion is well-nigh absurd. His only hope of salvation from an ever-deepening mental slough is, that the after-cares of practice may rouse him into action in some given direction. If, however, the question pursued be one to tax the utmost powers of the mind, so intimately interlaced are the many branches of science which contribute to a knowledge of the healing art, that we perceive the pre-eminent study of one portion act as a lever to raise the standard of knowledge of all the rest. Such a mode of study, indeed, rightly followed, most fully develops the powers of the mind, and necessitates a more intimate acquaintance with all that bears immediately or remotely upon the subject studied. Thus it is that speciality of study—a true speciality, as we have already stated,—is the life's blood of the theory and practice of physic. It is by many men concentrating their attention on solitary and different points that the advance of medical and of all other sciences is secured, not by all men devoting their attention to every point. What would have been the position of physic had we had to depend for its advancement—for the improvement of our knowledge of diseases of the heart, the lungs, the bowels, the brain, or of any and every part of the frame,—upon the efforts of those who are assumed to study with equal attention any and every form of disease, and not upon those who devote themselves specially to this or that form?

The men who attach themselves to some speciality in the study of the theory or practice of medicine, are the specialists of pure blood; but it by no means follows that the object of that study, its results, and the career of the man should be the same. Apart from the love of study *per se*, one man centres his hopes on the attainment of scientific fame, and looks fondly forward to the time when he may attach F.R.S. to his name: another man, devoted to a speciality which takes him to the bedside, where alone it can be developed, hopes by the honest and hard pursuit of his peculiar study to attain to an honourable competence for himself and family. Is it for the man, however, who seeks for scientific honour above all things, and who too commonly neglects the every-day world about him, to strike his more provident brother over the face and say, "Thou art less honourable than I"? Surely not. Each has chosen his own course, each has fixed his aim upon a different object, each has fought honourably his fight, each has contributed his mite to the benefit of mankind; but if the gift of the one has rendered more immediate good and brought more tangible returns than that of the other, is it for the less fortunate one in the world's eye to wrap himself up in an abstract and offensive purism, and say, "I am worthier than thou"?

The specialists who rise to eminence in the world's eye, and cull largely of the substantial favours which follow thereupon, constitute a small although glittering item among the true specialists found in the profession. It is only in great towns or the metropolis where the circumstances favourable to the existence of specialist practice are found; but the principle which prompts to the exercise of specialities does not on that account fall abortive to the ground in the country. Specialists both in theory and practice are, happily for the profession, broadcast over the land; and one need only look over the pages of our journals or the lists of medical books to obtain a knowledge of the rich gain to medical science which is perpetually arising from the pursuit of special studies in different branches of the theory and practice of medicine in every district of the kingdom. But in our great towns, and above all in the metropolis, the specialist in practical medicine meets with the combination of circumstances which foster best his peculiar studies and his success. It is there only, and most commonly in the wards of our hospitals, that the opportunities are found for extensive research. And what follows? Years of hard, relentless labour, clinical and literary, until the ear and (in the end) the confidence, of the profession are secured; and these once secured, what again follows? Our specialist's aid in his special branch of study is sought by his fellow medical men; the public very wisely follow suit; a stream of the cases to which he has chiefly given attention sets in upon him; the stream swells by degrees, and in the end predominates largely over all other forms of disease coming under his care; and our specialist stands before the world as a specialist in the most restricted acceptance of the term, but a specialist who has honourably struggled for, and nobly won, his speciality, and whose speciality has equally benefited the profession, the public, and himself. Here, then, we have a criterion of the specialist of true blood—one in whom the confidence both of the profession and the public converge, and thankful we are for our own and for physic's sake that there are several of these men among us.

That there are those who use the rightly acquired fame of the true specialist as a stalking-horse, and seek under its cover to impose upon the ignorance or the credulity of the public, is not to be wondered at, unless we assume a standard of morality in human nature which the world has not yet seen, nor is likely ever to see. Neither is there matter of marvel that these individuals —of whom we have borrowed a sketch from the *Paris-Médecin*— should in the opinion of many inflict disgrace upon specialist physicians and specialities in the practice of physic. But such a conclusion arises, we submit, entirely from a misapprehension of the subject—a misapprehension which, whenever it exists, leads to this grievous result, that in condemning thoughtlessly, and without due reservation, specialists and specialities in medicine, a blow is struck at one of the most vital points in the development of theoretical and practical physic—a point than which none is more essential to the well-being of medical science.

Such evils as appertain to specialist practice and specialities in medicine may be readily distinguished from the good which belongs to them, and which infinitely outgrows the evil. We do our duty best by depicting the character of the true specialist, and furnishing a criterion of that character. If there be any who think that it is practicable by trenchant means to do away with the tribe of spurious specialists, we shall not quarrel with the thought, but simply hint, lest they should throw efforts away which might be more usefully employed, that *It's a far cry to Utopia.*

---

